# Shifts in the fluorescence lifetime of EGFP during bacterial phagocytosis measured by phase-sensitive flow cytometry

**DOI:** 10.1038/srep40341

**Published:** 2017-01-16

**Authors:** Wenyan Li, Kevin D. Houston, Jessica P. Houston

**Affiliations:** 1 Chemical and Materials Engineering, New Mexico State University, Las Cruces, NM, 88003, USA; 2 Chemistry and Biochemistry, New Mexico State University, Las Cruces, NM, 88003, USA

## Abstract

Phase-sensitive flow cytometry (PSFC) is a technique in which fluorescence excited state decay times are measured as fluorescently labeled cells rapidly transit a finely focused, frequency-modulated laser beam. With PSFC the fluorescence lifetime is taken as a cytometric parameter to differentiate intracellular events that are challenging to distinguish with standard flow cytometry. For example PSFC can report changes in protein conformation, expression, interactions, and movement, as well as differences in intracellular microenvironments. This contribution focuses on the latter case by taking PSFC measurements of macrophage cells when inoculated with enhanced green fluorescent protein (EGFP)-expressing *E. coli*. During progressive internalization of EGFP-*E. coli*, fluorescence lifetimes were acquired and compared to control groups. It was hypothesized that fluorescence lifetimes would correlate well with phagocytosis because phagosomes become acidified and the average fluorescence lifetime of EGFP is known to be affected by pH. We confirmed that average EGFP lifetimes consistently decreased (3 to 2 ns) with inoculation time. The broad significance of this work is the demonstration of how high-throughput fluorescence lifetime measurements correlate well to changes that are not easily tracked by intensity-only cytometry, which is affected by heterogeneous protein expression, cell-to-cell differences in phagosome formation, and number of bacterium engulfed.

The human immune system is challenged by a vast number of bacterial pathogens, and the number continues to grow as species evolve. The development of methods to monitor and control host-pathogen interactions is thus essential from a system response level down to the single cell level. Among many different ways in which immune cells respond to invading bacterial cells is the process of phagocytosis. Phagocytosis is well-characterized and has been readily observed *in vitro* with mammalian and bacterial cell experiments^1^,^2^. Immune cells such as macrophages, eosiniphils and neutrophils can phagocytize bacterial cells, and macrophage cells play the major role in capturing and engulfing bacteria cells for elimination^3^. Internalization steps involve the development of membrane-like vesicles called phagosomes, that confine bacteria for further degradation^4^,^5^,^6^. The process is regulated by the macrophage cells in a functional polarization state that enables phagosomal pH regulation^7^,^8^,^9^. In fact, acidification of the phagosome is a mechanism that contributes to bacterial cell death^10^,^11^. Phagocytic acidification creates a harsh environment to prevent the invading bacterial cell from post-engulfment survival; it has been reported that the pH decreases from 7.0 to ~4.5 as the phagosome matures^12^.

The observation of phagocytosis as well as phagocytic acidification has been achieved with the aid of fluorescent dyes and proteins. Exogenous fluorophores, chemical dyes, or fluorescent proteins may be used to label phagocytic subunits at the single cell level. For example, fluorescence labels that bind to membrane embedded proton-pump proteins help visualize early phagosome states when membrane fusion events take place^13^. Other examples include expression of fluorescent proteins fused to the LC-3 protein during the late phagosome-lysosome-autophagosome merging processes^2^. It is also common to fluorescently label the target pathogen; Xu and colleagues studied phagocytic activity by expressing fluorescent proteins in bacteria which were subsequently introduced into mammalian cultures^14^. Similarly, Oh *et al*. dual-labeled *Mycobacterium avium* with both pH-sensitive and pH-insensitive dyes to measure the phagosomal pH during the phagocytosis process^15^. Other pH indicator dyes are used that spectrally shift with microenvironmental pH variation^3^,^15^ or fluoresce in acidic environments (i.e. emission positively correlates with increased acidity). Examples of pH-indicator dyes include laboratory-synthesized pH sensors (e.g. M) and commercially available fluorophores (e.g. pHrodo)^16^,^17^.

Fluorescent probes aid in the ability to understand the stages of the phagocytic process yet challenges remain because phagosome formation is multi-step with proteins that function in concert. Accordingly, pH shifts may be hard to validate with spectrally shifting dyes if there is cell-to-cell heterogeneity during phagocytosis (i.e. nonuniform fluorescence emission) or when the fluorescence emission is measured at a range of continuous times as pH changes. With pH-indicator dyes the total fluorescence emission is not as quantitative because the total increase in fluorescence might be a result of the gradual phagosomal acidification or the increase in the number of phagosomes formed^16^,^18^,^19^,^20^,^21^,^22^. Moreover, bacterial species might escape the phagosome owing to their ability to withstand the acidic microenvironment, as shown with Förster Resonance Energy Transfer (FRET) experiments^23^. Thus phagocytic-based research is increasingly dependent on multiple fluorescence labels, full spectral measurements, complex data analysis, efficient sample-screening, fluorescence ratio-metric methods and combinations of spectrofluorometry, fluorescence microscopy and flow cytometry^9^,^13^,^14^,^15^,^17^,^24^,^25^.

It would be beneficial to add a measurement of the fluorescence lifetime to studies of phagocytosis and acidification because fluorescence decay times contribute to a quantitative analysis of fluorophore presence. Fluorescence lifetimes often shift with changing intracellular microenvironments (i.e. pH, temperature)^26^,^27^. Additionally the average time a fluorophore spends in the excited state is independent of fluorescence intensity; brightness, or intensity, is influenced by factors such as fluorophore concentration, quantum yield, quantum efficiency and instrumentation artifacts. With flow cytometry, the use of fluorescence excited state decay parameters is particularly important because results are not influenced by spectral spillover into different fluorescence channels^28^, or the heterogeneity of fluorescence expression across single a cell (i.e. nuclear vs. cytoplasmic fluorescence emission).

In this work, we present phase-sensitive flow cytometry (PSFC) as a tool to detect phagocytosis through the measurement of the fluorescence lifetime on a cell-to-cell basis. We measure the fluorescence lifetime of enhanced green fluorescent protein (EGFP) expressed in bacterial cells when the cells are introduced into monolayers of macrophage cell cultures (RAW264.7). Using a combination of fluorescence microscopy and PSFC we are able to observe the internalization of EGFP-expressing *E. coli* (EGFP-*E. coli*) by the macrophage cells and detect average fluorescence lifetime changes during this process. EGFP is pH sensitive with reported fluorescence lifetime values that change with intracellular pH^26^,^29^. Measuring the decay kinetic properties of EGFP not only demonstrates a new approach for correlating photophysical quantities to phagosome formation and acidification but also provides a metric that is not affected by the heterogeneity in the number of bacterial cells confined per-macrophage cell. Additionally, the cytometry approach provides a measurement of fluorescence lifetimes for large, statistically significant populations of bacteria-engulfed macrophage cells.

## Results

### Flow cytometry measurements of macrophage cells and EGFP-expressing *E. coli*

**Figure 1:**
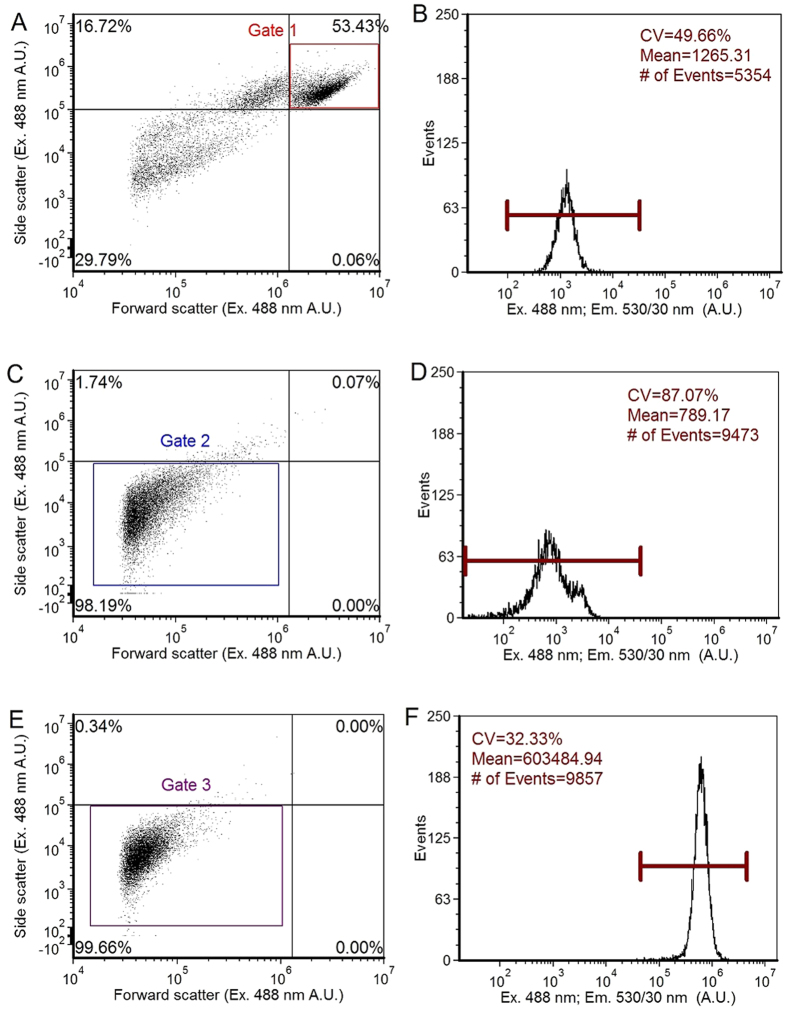
Flow cytometry results. (**A**), (**C**) and (**E**) dot plots (n = 10000 cells) of side scatter versus forward scatter of RAW264.7 macrophage cells, *E. coli* and EGFP-*E. coli*, respectively. (**B**), (**D**) and (**F**) fluorescence intensity (green channel 530/30 nm) histograms of RAW264.7 cells within Gate 1 (red box) in (**A**), *E. coli* within Gate 2 (blue box) in (**C**) and EGFP-*E. coli* within Gate 3 (purple box) in (**E**), respectively. Low fluorescence intensities of RAW264.7 cells (mean = 1265.31; and CV = 49.66%) and *E. coli* (mean = 789.17; and CV = 87.07%) compared to EGFP-*E. coli* (mean = 603484.94; and CV = 32.33%) indicate that the cells with no EGFP are measuring autofluorescence.

RAW264.7, a mouse macrophage cell line, *E. coli*, and EGFP-expressing *E. coli* (EGFP-*E. coli*) were analyzed by flow cytometry (Accuri, BD Biosciences, New Jersey, USA.) and standard measurements including total fluorescence intensity, side scatter intensity, and forward scatter intensity were collected. Side scatter vs. forward scatter dot plots are provided for RAW264.7, *E. coli*, and EGFP-*E. coli* cells (Fig. 1A,C,E, respectively). Fluorescence intensity was measured (green channel emission at 530/30 nm) in each cell line used in this study (Fig. 1B,D,F, respectively). These data show that scattered light intensity is higher for the relatively larger RAW264.7 cells compared to the scattered light intensity of smaller *E. coli* and EGFP-*E. coli* cells indicated by events located in the top right quadrant for RAW264.7 cells and events located in the bottom left quadrant for *E. coli* and EGFP-*E. coli* cells. The average fluorescence intensity of EGFP-*E. coli* cells was 3 orders of magnitude higher than the autofluorescence intensity of *E. coli* and RAW264.7 cells. Gating analysis revealed that 98% of EGFP-*E. coli* cells expressed EGFP.

### Fluorescence microscopy imaging of phagocytosis

**Figure 2:**
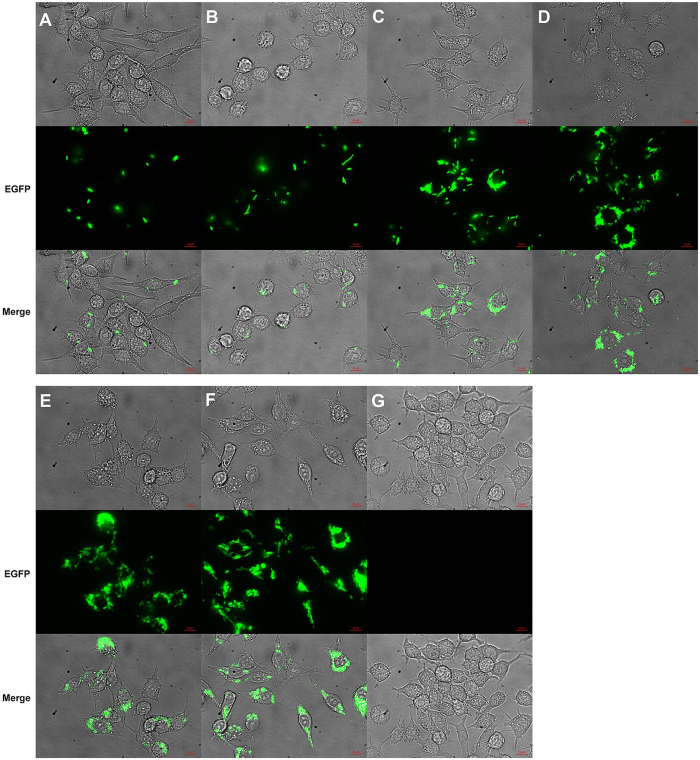
Fluorescence microscopy results. (**A**–**F**) RAW264.7 cells images taken with bright field as well as fluorescence at 530-nm. The images panels (A through F) depict infection occurring by EGFP-*E. coli* at different time points: 5, 30, 60, 90, 120 or 180 min, respectively. Panel (G) provides bright field and fluorescence images of RAW264.7 cells not treated with the EGFP-expressing *E. coli*.

Epifluorescence microscopy was used to image RAW264.7 cells after inoculation with EGFP-*E. coli* at multiple time points. Representative images of RAW264.7 cells captured at six time points post-inoculation from 5 to 180 min (Fig. 2A–F) and RAW264.7 cells that were not inoculated with EGFP-*E. coli* (Fig. 2G) show internalized EGFP-*E. coli* at 5 min post-inoculation with saturation of fluorescence emission between 60–90 min. While these data demonstrate increased EGFP-*E. coli* phagocytosis over time, the accumulation of EGFP-*E. coli* was not the same between cells indicated by differences in fluorescence intensity among individual cells.

### Fluorescence lifetime measurements with phase-sensitive flow cytometry

**Figure 3:**
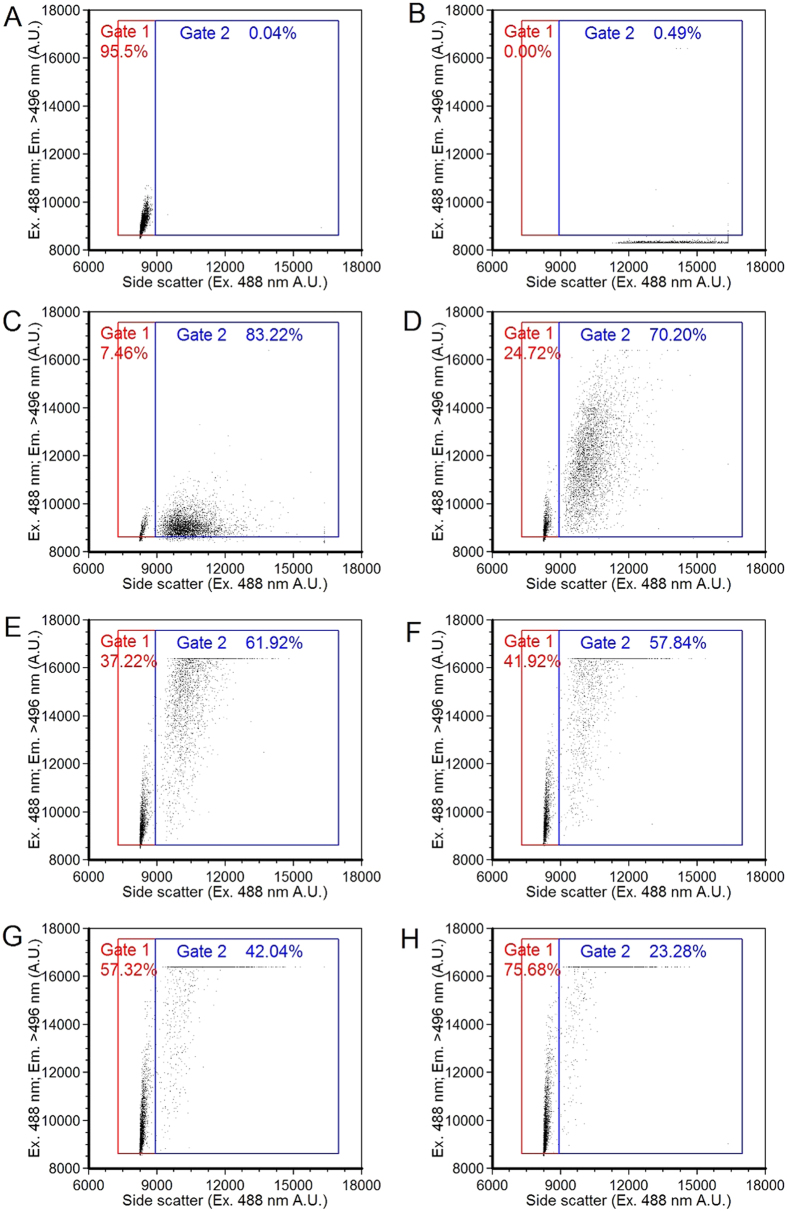
Measurements of the mean fluorescence intensity with a phase-sensitive flow cytometer. Cytometric dot plots (n = 5000 cells) include (**A**) EGFP-*E. coli* cells counted without macrophage cells, (**B**) non-infected RAW264.7 cells, and (**C**) through (**H**) displaying RAW264.7 cell counts infected by EGFP-*E. coli* at times 5, 30, 60, 90, 120 and 180 min, respectively. Within Gate 1 from (**A**) through (**H**) mean fluorescence intensities are 9085 (CV = 3.33%; n = 4774), N/A, 8938 (CV = 3.03%; n = 373), 9062 (CV = 4.77%; n = 1236), 9494 (CV = 7.8%; n = 1861), 9691 (CV = 8.33%; n = 2096), 9704 (CV = 9.73%; n = 2866) and 9585 (CV = 9.84%; n = 3784), respectively; and mean SSC intensities are 8395 (CV = 1.19%), N/A, 8386 (CV = 1.22%), 8351 (CV = 1.06%), 8365 (CV = 1.22%), 8362 (CV = 1.12%), 8360 (CV = 1.14%) and 8341 (CV = 1.0%), respectively. Within Gate 2 from (**A**) through (**H**) mean fluorescence intensities are N/A, N/A, 9082 (CV = 4.57%; n = 4161), 11802 (CV = 12.84%; n = 3510), 15145 (CV = 10.68%; n = 3096), 15630 (CV = 9.28%; n = 2892)), 15894 (CV = 7.8%; n = 2102) and 15910 (CV = 7.69%; n = 1164), respectively; and and mean SSC intensities are N/A, N/A, 10361 (CV = 7.93%), 10372 (CV = 6.99%), 10628 (CV = 7.31%), 10722 (CV = 7.88%), 10887 (CV = 8.79%) and 10737 (CV = 9.15%), respectively.

Phase-sensitive flow cytometry (PSFC) was performed with a modified FACSVantage™ flow cytometer. The PSFC measurements included fluorescence intensity, side-scatter intensity, and fluorescence lifetime. Representative side-scatter vs. fluorescence intensity dot plots for EGFP-*E. coli*, RAW264.7 cells, and RAW264.7 cells at various time points post-inoculation with EGFP-*E. coli* are shown with two gates (Gate 1, red; and Gate 2, blue). The gates emphasize the differences in fluorescence and side-scattered light emitted associated with each cell group (Fig. 3). For example, the dot plot obtained for EGFP-*E. coli* shows that measurements of fluorescence and side-scatter intensity associated with this cell type are within Gate 1 (red box, Fig. 3A), while non-inoculated RAW264.7 cells that have relatively low autofluorescence intensity yet higher side-scatter intensity when compared to EGFP-*E. coli* are included in Gate 2 (blue box, Fig. 3B). The dot plot for side-scatter vs. fluorescence intensity measurements for RAW264.7 cells 5 min post-inoculation have higher side-scatter intensity (due to a larger size relative to EGFP-*E. coli*) and have a significantly higher fluorescence intensity when compared to non-inoculated RAW264.7 cells (Fig. 3C). These data suggest that the fluorescence intensity of the EGFP-*E. coli*-inoculated RAW264.7 cells is due to internalization of EGFP-*E. coli*. At the earlier time points, low numbers of EGFP-*E. coli* have been internalized by a small percentage of the RAW264.7 cells. With increasing time post-inoculation (>30 min), the number of EGFP-*E. coli* inside the RAW264.7 cells is increased resulting in higher mean fluorescence intensities (Fig. 3D). Data demonstrating a consistent increase over time in the mean fluorescence intensity of the EGFP-*E. coli*-inoculated RAW264.7 cells is shown (Fig. 3E–H). This accumulation over time reached saturation at 60 min post-inoculation and provided a measurable EGFP fluorescence emission for phase-sensitive flow cytometry measurements. In summary the number of the engulfed EGFP-*E. coli*, measurable by the increased mean fluorescence intensity using flow cytometry was positively correlated with post-inoculation times from 5–60 min.

**Figure 4:**
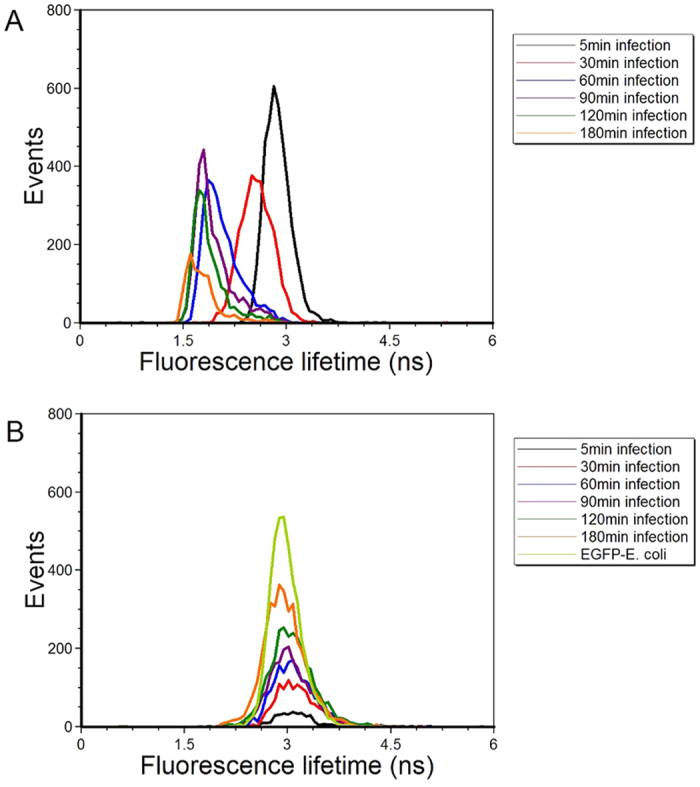
Measurements of the mean fluorescence lifetimes with a phase-sensitive flow cytometer. In panels (A) are fluorescence lifetime histograms of the average fluorescence lifetime of EGFP when accumulated via *E. coli* into RAW264.7 cells at 5, 30, 60, 90, 120 and 180 min. Each histogram trace is color coded to indicate an increasing time point. Panel (B) provides the fluorescence lifetime histograms in which the fluorescence lifetime of EGFP was measured when expressed in *E. coli* when not in contact with the macrophage cells. For experimental control, the measurements were taken at the same time points as that for the infection treatments.

To demonstrate the feasibility of using fluorescence lifetime measurements by PSFC for monitoring phagocytosis, RAW264.7 cells were inoculated with EGFP-*E. coli* and the fluorescence lifetime of EGFP was measured using a modified FACSVantage™ flow cytometer. Prior to measuring fluorescence lifetime for RAW264.7 cells, the fluorescence instrument was calibrated using Flow Check Fluorospheres™ (Beckman Coulter); these microspheres have an average fluorescence lifetime of 7 ns (data not shown)^30^. Histogram representation of the fluorescence lifetime distributions for RAW264.7 cells 5 to 180 min post-inoculation with EGFP-*E. coli* and EGFP-*E. coli* cells are provided in Fig. 4. The fluorescence lifetime distributions were generated using gated data corresponding to Gates 2 and 1, respectively. The fluorescence lifetimes of the phagocytosed EGFP-*E. coli* are decreased relative to the fluorescence lifetime measured in EGFP-*E. coli* cells (Fig. 4B). Table 1 provides the results obtained from these experiments as an average of each population (n = 5000 cells) from 4 independent experiments. Additionally, the average fluorescence lifetime of non-phagocytized EGFP-*E. coli* measured at similar time points for controlled experimental comparison remained between 3.1 and 3.3 ns. In contrast, when fluorescence lifetime was measured in RAW264.7 cells at 5, 30, 60, 90, 120 and 180 min post inoculation with EGFP-*E. coli* cells, a consistent decrease of 3, 2.8, 2.3, 2.1, 2 and 2-ns was observed, respectively.

## Discussion

Phagocytic acidification is a well-studied defense mechanism that macrophage cells employ, and the detection of a lowering pH is challenging to reliably observe. Although a variety of pH sensors^12^,^16^,^31^ exist, most of the studies performed use fluorescence microscopy and the accumulation of fluorescence in cells is heterogeneous and difficult for large scale measurements such as flow cytometry. In this contribution we demonstrate how PSFC can be used to detect fluorescence lifetime-dependent changes and that those changes correlate to gradual bacteria-macrophage interactions.

The fluorescence lifetime is determined by the decay pathway of an excited fluorophore and is independent of fluorescence intensity. Therefore the fluorescence lifetime is often used to add a quantitative metric to fluorescence microscopy or flow cytometry because it is not dependent on the concentration of the fluorophore, the quantum efficiency, instrumentation artifacts, and can overcome spectral overlap and autofluorescence background issues. What is also interesting about the fluorescence lifetime of many molecules is that it is affected by a changing microenvironment such as pH, temperature, presence of quenchers and other energy transferring molecules. By exploiting what is known about the photophysical properties of EGFP, specifically how fluorescence lifetime shifts with pH^26^, we are able to perform a correlative study to compare lifetimes during intracellular transformations where pH is known to change as compared to cells not undergoing the phagocytic process. In order to control for the possibility of other intracellular events (e.g. presence of quenchers^32^ and cellular polarity^33^) affecting the phase-resolved signal, we measured total fluorescence emission and selected clonal cell populations known to not exhibit polarization phenotypes^9^.

We found that the non-confined EGFP-*E. coli*, did not have an average fluorescence lifetime shift from reported fluorescence lifetimes of EGFP in the literature (i.e. 3.1–3.3 ns)^29^,^34^,^35^. Alternatively, when the fluorescent protein expressing *E. coli* were engulfed by RAW264.7 cells, and trapped in the membrane-like vacuoles where a decrease of pH is known to occur, the fluorescence lifetime shifted by 1 ns. The change in fluorescence lifetime was correlated to the phagocytic process whereby hydrogen ions are reported to become more abundant. In this controlled setting a rationale is that the fluorescence lifetime shift occurs when the EGFP chromophore is exposed to protons that affect the decay kinetic properties. We found that the average fluorescence lifetime of EGFP dropped from 3 ns to 2 ns as infection time progressed from 5 min to 180 min.

In summary, these results demonstrate that intracellular accumulation of *E. Coli*, which express EGFP inside of macrophage cells, is correlated to a decrease in the EGFP fluorescence lifetime when compared to control cells. Our results show that earlier engulfed EGFP-*E. coli* have shorter mean fluorescence lifetimes when compared to later engulfed EGFP-*E. coli* between 5 to 120 min. We believe this presents an interesting step toward the use of EGFP fluorescence decay kinetics as a pH indicator inside of cells. Phagocytosis is known to involve localized pH changes, and while it is probable that this mechanism be studied at a cytometry throughput with pH-sensor dyes, the process of engulfment itself is heterogeneous. The results presented are a first step toward obtaining quantitative cell-to-cell statistics of subtle, intracellular pH shifts. Furthermore our technique can be integrated with phasor^36^ or multi-frequency lifetime approaches^37^ to resolve multiple fluorescence lifetime components within individual cells. Future analysis with multiple-phase information will allow us to further validate the ratio of engulfed bacteria during early inoculation stages to bacteria-mammalian cell interactions at late stages.

## Methods

### Plasmid transformation and *E. coli* cell culture

Introduction of EGFP into bacteria cultures was performed with an EGFP mutant DNA plasmid constructed by Dr. Miho Suzuki (generously gifted from Saitama University, Japan). The One Shot BL21(DE3) kit (Life Technologies, CA, USA) was used for standard bacterial transformation. The transformed *E. coli* bacteria and a control culture were grown overnight on a LB agar plate with 50 *μ*g/mL ampicillin in an incubator at 37 °C and then stored in a refrigerator at 4 °C for later use.

### Macrophage cell culture and bacterial infection

The mouse macrophage cell line, RAW264.7 was obtained from ATCC^®^ and standard culture techniques were applied. Cells were incubated with Dulbecco’s Modified Eagle Medium (DMEM) with 10% Fetal Bovine Serum (FBS) at 37 °C and 5% CO_2_ in a humidified environment. For bacterial inoculation into macrophage cells we followed similar protocols whereby phagocytosis was observed^14^. Colonies of EGFP expressing *E. coli* were transferred from the LB agar plate to a 14 mL round bottom tube filled with 3 mL of LB broth and 50 *μ*g/mL ampicillin medium for 20 hours at 37 °C and 225 rpm. RAW264.7 cells were plated on a 12-well plate to study uptake of bacteria at different time points when at 80 to 90% confluence. *E. coli* bacteria and macrophage cells were counted separately to control the multiplicity of infection (MOI) for each well at approximately 10. Required volumes of *E. coli* suspensions were transferred for centrifugation (5 min at 4000 rpm). The cell pellets were suspended with pre-warmed DMEM + 10% FBS media and equally distributed into each well of macrophage cells. The cells were then incubated and at 5, 30, 60, 90, 120, or 180 min, and for each separate time point the inoculated cell population was trypsinized, suspended in PBS, and collected for flow cytometry measurements. Three independent experiments were performed and data shown are representative of all replicates. A similar experiment was performed using 35 mm and 23 mm diameter round bottom glass dishes in order to culture cells for direct fluorescence microscopy imaging and avoid trypsinization and removal.

### Fluorescence microscopy and standard flow cytometry

A standard epifluorescence microscope was used to image *E. coli* bacteria introduced into the macrophage RAW264.7 cell cultures. The Zeiss Axiovert S100 microscope (Carl Zeiss, Oberkochen, Germany) is configured with a 450–490 nm band pass filter for excitation and a 515–565 nm filter for fluorescence emission. Owing to the size of the *E. coli* bacterium relative to macrophage RAW264.7 cell, we used a 63-magnification objective oil immersion lens. Flow cytometry was performed with the AccuriC6™ flow cytometer (BD Biosciences, CA, USA). Cytometry was performed with this instrument to evaluate the mean fluorescence intensity (530/30-nm band pass filter) and side scatted light using *E. coli* cells and *E. coli* expressing EGFP cells.

### Phase-sensitive flow cytometry instrument development

**Figure 5:**
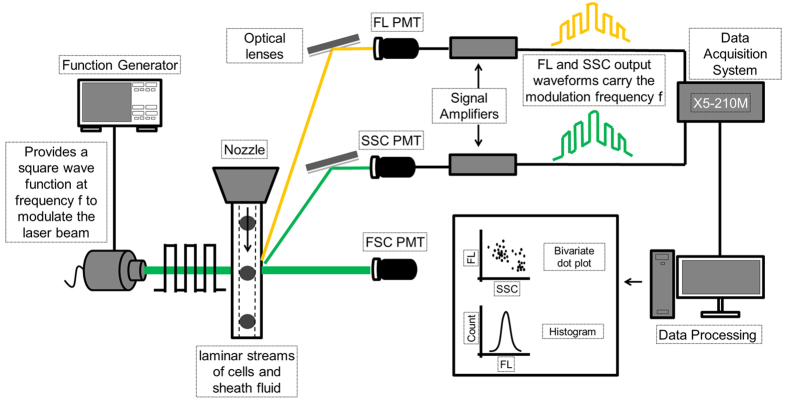
Depiction of the phase-sensitive flow cytometer. Departing from the nozzle to a digitally modulated laser beam are cells through a pressure driven fluidic system. SSC and FL PMT signals are transformed to ‘cytometric waveforms’ by the data acquisition system. Output waveforms are fed to the data analysis software for SSC and FL light intensities and fluorescence lifetimes calculation.

A frequency-domain flow cytometry system was developed by modifying a commercial instrument (FACSVantageTM SE, Becton Dickinson, NJ, USA) as diagramed in Fig. 5. This cytometric approach adapts time-resolved fluorescence spectroscopy to a flow cytometer. To be noted here is the idea that phase-sensitive measurements herein measure the kinetics of fluorescence excited states as opposed to time-resolved cytometry approaches in which intensity-based cytometry measurements are tracked over time to observe changes across cell populations as they pass through the excitation source^38^,^39^,^40^. A solid-state laser diode (488 nm, Vortran, CA, USA) was digitally modulated at 25 MHz with a function generated digital square wave (Tektronix AFG3102, Tekronix, OR, USA). Cells were passed through the pressure driven fluidics and through the focused laser beam waist in single file at an approximate rate of 1000 cells/second. A photomultiplier tube (PMT, R12829 PMTs, Hamamatsu, Japan) collected excitation light scattered in the side direction (“side scatter channel”, SSC) with a 488-nm +/− 5-nm band pass filter. A second, similar PMT collected fluorescence emission (FL) using a 496 nm long pass filter. A data acquisition module X5–210 M (Innovative Integration, CA, USA) with a sampling rate of 250 MSPS and signal amplifiers (DC-100, Advanced Research Instruments, OR, USA) was used to digitize the PMT signals for on-board calculation of average fluorescence lifetime values. The fluorescence lifetime values were obtained with FPGA processing using a Goertzel algorithm (described below)^41^,^42^, which extracts the phase difference between the SSC and FL for each cell. The phase difference is proportional to the fluorescence lifetime^43^. Calibration of the fluorescence lifetime was performed with Flow-Check^TM^ Fluorospheres (Beckman Coulter, CA, USA), which have a known fluorescence lifetime of 7 ns. After calibration, all settings remained the same for each proceeding experiment.

### Computational approaches for frequency-domain measurements

Discrete Fourier transform (DFT) is commonly used to convert a time function into a spectrum of frequencies. Instead of computing a range of frequencies, the Goertzel algorithm derived from DFT is able to exclusively resolve single or multiple frequencies of interest. The DFT equation of a vector x[n] of length N is defined by


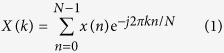


The derivation of the Goertzel algorithm begins with multiplying both sides of (1) by


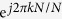


where


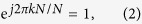


and returns


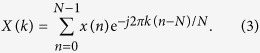


y_k_(m) as a filter function is then defined by


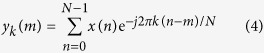


where at m = N


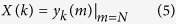


Through z-transform and infinite impulse response (IIR), yk(m) is further derived to the equations below


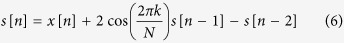


and


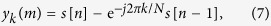


therefore


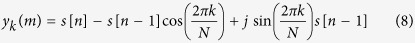


Equations (6), (7) and (8) show that the real and imaginary parts of y_k_(m) are constructed by the components of s[n], s[n − 1], s[n − 2], cos(2*π*k/N) and sin(2*π*k/N). Hence, the computational approach taken to measure the fluorescence lifetime firstly pre-calculates the cosine and sine terms. The angular frequency, *w*, is found where *k* is a coefficient related to the modulation frequency (target frequency) and sampling rate, *N* is the number of data samples, *a, b* and *c* are coefficients calculated based on *k* and *N*.


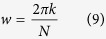



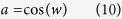



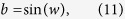


and


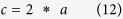


Secondly, after obtaining the angular frequency at the sampling rate and determining the sine and cosine at this frequency, s[n], s[n − 1] and s[n − 2] are initialized to zero and are termed as *S*_0_, *S*_1_ and *S*_2_. These three initialization variables are encoded to initiate a rapid loop that will lead to the calculation of the real and imaginary components of the frequency domain output signal. The loop sequence executed by the digital signal processing is provided by


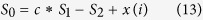



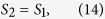


and


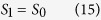


where *x(i*) represents a vector of data points of any waveform and *i* ranges from *1* to *N*. At the end of the loop where m = N, X(k) is equal to y_k_(m) and therefore the frequency of interest is filtered out.

Lastly, the phase content (*θ*) at the modulation frequency is calculated from the real and imaginary components of y_k_(m) as shown below where


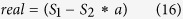



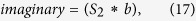


and


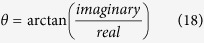


The average fluorescence lifetime, *τ*, is computed by


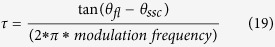


## Additional Information

**How to cite this article:** Li, W. *et al*. Shifts in the fluorescence lifetime of EGFP during bacterial phagocytosis measured by phase-sensitive flow cytometry. *Sci. Rep.* **7**, 40341; doi: 10.1038/srep40341 (2017).

**Publisher's note:** Springer Nature remains neutral with regard to jurisdictional claims in published maps and institutional affiliations.

## Figures and Tables

**Table 1:** Results of mean fluorescence lifetimes of EGFP expressed in *E. coli*.

Time (min)	5	30	60	90	120	180	Control
Infected RAW 264.7, FL (ns)	3.0 +/− 0.2	2.8 +/− 0.13	2.3 +/− 0.19	2.1 +/− 0.14	2.0 +/− 0.13	2.0 +/− 0.16	not measurable
Non-confined *E. coli*, FL (ns)	3.1 +/− 0.08	3.2 +/− 0.17	3.3 +/− 0.17	3.1 +/− 0.22	3.1 +/− 0.15	3.1 +/− 0.13	3.1 +/− 0.17

The standard deviations are for repeated (n = 4) populations (n = 5000) of cells counted with a phase-sensitive flow cytometer. Samples were measured at different time points after the macrophage RAW 264.7 cells were treated with bacteria. The control samples were RAW 264.7 cells without bacteria treatment and *E. coli* populations not added to macrophage cell cultures.
